# Exercise intervention protocol in children and young adults with cerebral palsy: the effects of strength, flexibility and gait training on physical performance, neuromuscular mechanisms and cardiometabolic risk factors (EXECP)

**DOI:** 10.1186/s13102-021-00242-y

**Published:** 2021-02-26

**Authors:** Pedro Valadão, Harri Piitulainen, Eero A. Haapala, Tiina Parviainen, Janne Avela, Taija Finni

**Affiliations:** 1 Neuromuscular Research Center, Faculty of Sport and Health Sciences, University of Jyväskylä, Jyväskylä, Finland; 2 Department of Neuroscience and Biomedical Engineering, Aalto University, Espoo, Finland; 3 Institute of Biomedicine, School of Medicine, University of Eastern Finland, Kuopio, Finland; 4 Centre for Interdisciplinary Brain Research, University of Jyväskylä, Jyväskylä, Finland

**Keywords:** Cerebral palsy, Strength, Flexibility, Gait, Training, Neuromuscular, Cardiometabolic

## Abstract

**Background:**

Individuals with cerebral palsy (CP) have problems in everyday tasks such as walking and climbing stairs due to a combination of neuromuscular impairments such as spasticity, muscle weakness, reduced joint flexibility and poor coordination. Development of evidence-based interventions are in pivotal role in the development of better targeted rehabilitation of CP, and thus in maintaining their motor function and wellbeing. Our aim is to investigate the efficacy of an individually tailored, multifaceted exercise intervention (EXECP) in children and young adults with CP. EXECP is composed of strength, flexibility and gait training. Furthermore, this study aims to verify the short-term retention of the adaptations three months after the end of the EXECP intervention.

**Methods:**

Twenty-four children and young adults with spastic CP will be recruited to participate in a 9-month research project with a 3-month training intervention, consisting of two to three 90-min sessions per week. In each session, strength training for the lower limbs and trunk muscles, flexibility training for the lower limbs and inclined treadmill gait training will be performed. We will evaluate muscle strength, joint flexibility, neuromuscular and cardiometabolic parameters. A nonconcurrent multiple baseline design with two pre-tests and two post-tests all interspaced by three months is used. In addition to the CP participants, 24 typically developing age and sex-matched participants will perform the two pre-tests (i.e. no intervention) to provide normative data.

**Discussion:**

This study has a comprehensive approach examining longitudinal effects of wide variety of variables ranging from physical activity and gross motor function to sensorimotor functions of the brain and neuromuscular and cardiometabolic parameters, providing novel information about the adaptation mechanisms in cerebral palsy. To the best of our knowledge, this is the first intervention study providing supervised combined strength, flexibility and gait training for young individuals with CP.

**Trial registration number:**

ISRCTN69044459, prospectively registered (21/04/2017).

## Background

### Cerebral palsy

Cerebral palsy (CP) describes a group of permanent disorders of the development of movement and posture, that are attributed to nonprogressive disturbances that occurred in the developing fetal or infant brain [1]. The spastic-type CP accounts for approximately 80% of all CP cases [2–4] and is characterized by hyper-resistance [5] which has three components: stretch hyperreflexia (i.e. velocity dependent involuntary activation), involuntary background activation and altered muscle-tendon mechanical properties. Muscle-tendon unit mechanical alterations [6–8] can cause severe reduction in joint range of motion (i.e. contractures) and lead to bone deformation [9–11]. The development of contractures does not seem to be caused solely by hyperreflexia [12, 13]; rather muscle inactivity and impaired muscle growth seems to be important factors [14, 15]. Furthermore, another debilitating symptom in CP is a decreased muscle strength [6, 16–18] that severely hinders the ability to perform tasks such as walking and climbing stairs [19–21]. These secondary clinical symptoms induce lower physical activity (PA) levels in people with CP [22, 23] and lead to higher levels of body adiposity and other cardiometabolic risk factors [16, 24, 25]. Therefore, although the initial brain lesion is nonprogressive in CP, secondary symptoms such as reduced muscle strength and joint range of motion (ROM) typically further deteriorate with time, increasing sedentarism and creating a cycle of inactivity and loss of function [26, 27]. Thus, well targeted therapeutic interventions to reduce these secondary problems and maintain motor function are in pivotal role to enable independent mobility in individuals with CP and thus support their lifelong health and wellbeing.

### Strength training for CP

Cross-sectional studies demonstrated that muscle strength had a high shared variance with gait and motor function in CP (r^2^ = 50–60% [28, 29];). Furthermore, the effectiveness of strength training complying with known training guidelines [25, 30, 31] to increase muscle strength in individuals with CP has been well demonstrated [32–40]. Nevertheless, these interventions have reported no changes [33, 35–38] or improvement in motor function [32, 34, 35]. This controversy seems to arise from two main reasons. Firstly, functional tasks involve a complex interaction between muscle strength, joint flexibility (i.e. maximum joint ROM) and motor coordination. Secondly, the neural and morphological adaptations are highly specific to the strength training methods [35, 41], which varied in these studies. A final remark is that every day functional tasks involve activation of several muscle groups spanning across joints. Thus, an adequate intervention should train all relevant muscles with naturalistic patterns of neural activation.

A major yet unproven concern is that strength training could increase hyper-resistance and thus would be an unadvisable intervention [42]. Only few strength training studies have measured components of hyper-resistance longitudinally and reported no changes after the intervention [33, 34, 37, 38]. Furthermore, the term “spasticity” is often used under varied definitions and assessment methods [43]. For example, the modified Ashworth Scale [44] utilized in two of these studies [33, 34] is a subjective assessment of resistance to passive movement [45]. Scholtes et al. [37, 38] evaluated hyperreflexia by assessing the presence of a catch (i.e. sudden increase in muscle tone) in response to a single fast (< 1 s) passive stretch, being also a subjective measure of the velocity-dependent resistance to passive movement. Both measurements fail to differentiate between the different components of hyper-resistance. Therefore, although it is generally accepted that strength training does not increase “spasticity” [46], no longitudinal study has reported an objective, valid and reliable measurement of hyper-resistance.

### Flexibility training for CP

Stretching is often utilized to prevent or treat loss of joint flexibility, however a recent systematic review pooling different stretch modalities (e.g. splint, serial casting, manual therapy) for different patient groups (e.g. CP, stroke, spinal cord injury) reported high-quality evidence that it does not have clinically relevant effects [47]. Passive stretching has the advantage of not restricting joint movement for a prolonged time as opposed to serial casting or splints, however, its effectiveness to increase joint flexibility in people with CP is unclear [48, 49]. Theis et al. [50] demonstrated that passive dorsiflexion stretching caused an acute increase in flexibility due to the elongation of both muscle and tendon. However, comprehensive longitudinal data is scarce. Theis et al. [51] showed an increase in dorsiflexion flexibility after a 6-week passive stretching intervention (4 days per week, 15 min per day). Zhao et al. [52] demonstrated that passive dorsiflexion stretching and active ankle joint movement within a 6-week intervention was able to increase fascicle length of both soleus (Sol) and medial gastrocnemius (MG). However, the study did not have a control group and the sample was composed of children, thus maturation may have been an intervening variable. The disparity between the limited scientific understanding of stretching effectiveness for CP and its widespread utilization must be addressed swiftly.

### Gait training for CP

The inability to dorsiflex the ankle joint during the stance phase (i.e. toe walking) and swing phase of gait (i.e. foot drop) are common problems in CP [53]. A recent literature review has concluded that gait training is safe, feasible and effective to improve walking ability in children and young adults with CP [54]. The incline treadmill setup seems particularly effective as it forces a greater ankle dorsiflexion in the swing phase and serves as a dynamic passive stretch for the ankle plantarflexors in the stance phase [55, 56]. Daily gait training with an inclined treadmill has been demonstrated to increase walking speed, dorsiflexion strength and active ROM, and reduce ankle stiffness in only four to six weeks [57, 58]. No changes in ankle passive flexibility were found in these studies, however the decrease in passive stiffness suggests that the muscle-tendon unit is adaptable, but further improvements in ROM may require a longer period of training. It is noteworthy that altering gait pattern and automatizing it for use in everyday over ground walking may require intense individualized feedback, so the person is aware or is at least guided towards the correct pattern (e.g. verbal feedback, biofeedback/augmented reality; [54]).

### Study purpose

The present study has three aims: 1) to investigate the effects of the EXECP intervention on the following variables: gait performance (primary outcome), physical activity level, lower limb muscle strength and joint flexibility, gross motor function, cardiometabolic risk factors, neuromuscular parameters for triceps surae muscle, cortical processing of proprioceptive stimuli evoked by passive ankle dorsiflexions, tibialis anterior (TA) cortico-muscular and intramuscular coherence; 2) to evaluate the retention of the adaptations induced by the intervention after three months; 3) to compare CP and typically developing (TD) individuals in all studied variables using group and matched (age/sex) individual data. The EXECP intervention is composed of a three-month individually tailored exercise intervention containing strength training targeting lower limb and trunk muscles, combined with stretching of diagnosed short muscles and gait training. The primary hypothesis is that the EXECP intervention will enhance gait performance by: a) increasing walking distance in the 6 min walking test; b) increasing ankle dorsiflexion during the swing and stance phase of gait; c) increasing maximal walking velocity, joint net moments and ranges of motion in the lower limb joints. The secondary hypothesis are that the EXECP intervention will: d) increase habitual PA; e) increase maximal isometric and concentric torque and rate of force development for the trained muscles; f) not affect cardiometabolic risk factors such as arterial stiffness, insulin resistance, blood lipids and body fat content; g) increase gross motor function measure (GMFM; [19]) score; h) increase lower limb joint flexibility of the trained muscle groups; i) decrease ankle plantarflexors average joint stiffness and increase joint energy (i.e. area under the torque-angle curve) during slow passive stretching; j) decrease antagonist muscle electromyography (EMG) during maximal voluntary isometric and concentric plantarflexion and dorsiflexion; k) not change spinal mechanisms related to hyperreflexia in the Sol muscle (i.e. Hoffmann-reflex (H-reflex), post-activation depression (PAD) ratio, tonic stretch reflex threshold (TSRT)); l) increase amplitude of cortical responses to proprioceptive stimulation, recorded with magnetoencephalography (MEG) in the primary somatosensory cortex; m) increase cortico-muscular coherence between cortical MEG and TA EMG signals; n) increase intramuscular coherence within TA muscle during a submaximal isometric dorsiflexion and during the swing phase of the gait cycle. No changes in any of the studied variables are expected during the 3-months control period, except if the participant is going through the growth spurt. After the 3-month maintenance period, the values of the studied variables are expected to be in between the control and post intervention values (project aim B). All study variables are hypothesized to show differences between the TD and CP groups (project aim C). It is expected that the comprehensive assessment battery of neuromuscular, brain, cardiometabolic and functional parameters will add valuable information about how people with CP adapt to exercise.

## Methods

### Study design

**Fig. 1:**
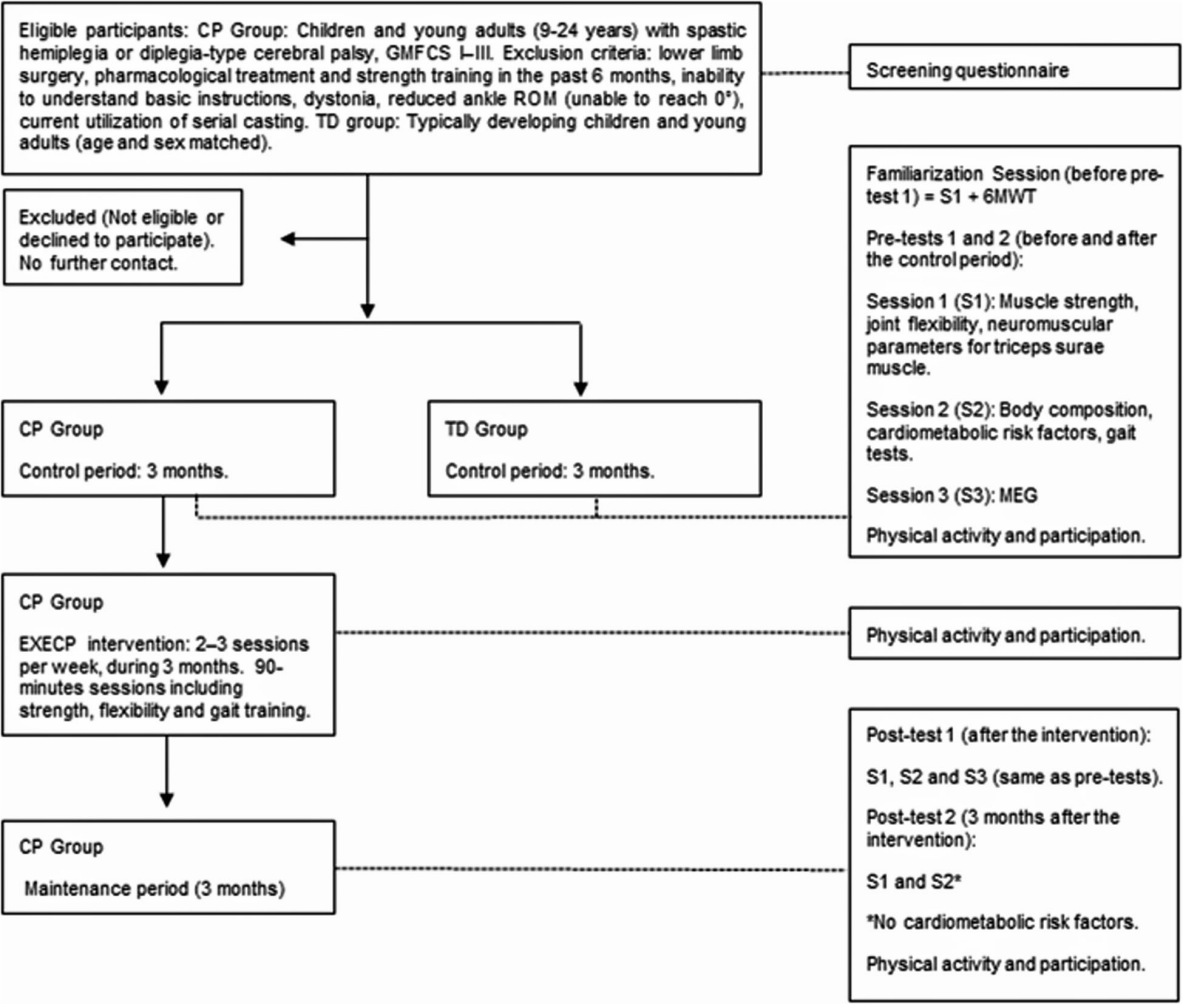
Flow chart for EXECP study. CP = cerebral palsy; TD = typically developing; GMFCS = gross motor function classification system; ROM = range of motion; 6MWT = six minutes walking test; MEG = magnetoencephalography

The present study utilizes a nonconcurrent multiple-baseline design [59, 60] composed of two pre-tests interspaced by three months (i.e. control period), followed by the three-month EXECP-intervention, and two post-tests performed immediately after and three months after the intervention (i.e. maintenance period; Fig. 1). The TD age and sex-matched control group will be tested in the two pre-tests without the intervention. The nonconcurrent multiple baseline design was selected over the randomized controlled trial (RCT) because the participants inside the inclusion criteria are expected to form a highly heterogeneous group regarding the studied variables (e.g. motor control, muscle strength, joint flexibility and gait mechanics), and randomly dividing the sample into two groups is not likely to result in two comparable groups. The chosen experimental design circumvents the expected high variability in the studied variables by allowing the subjects to be their own controls. Additionally, it allows performing the EXECP intervention all year-round, avoiding systematic seasonal influences to affect the study variables (e.g. vacations, seasons [61];). During the participation in the project, both groups will perform their normal activities (i.e. physiotherapy and/or sport activities), with the instruction of maintaining the number of sessions and activity type as stable as possible. Exceptionally during the intervention period, a reduction in the volume of the activities may be necessary to avoid overloading the participants. Any important deviations from the activities done during the control period will be reported. The first author will be responsible for all tests, and it is not possible to blind him to group allocation. Testing procedures were defined in detail to ensure consistency. The outcome data is quantitative and objective, with little possibility to suffer from observer bias. However, because the tester will obviously hope for better results in the Post-tests, no data analysis will be performed before the data collection is done (except for the flexibility tests). Thus, the tester will not have any knowledge of the results and will be focused on implementing the tests according to the protocol.

### Study sample and recruitment

Twenty-four individuals aged 9–24 years with spastic-type CP will be recruited predominantly in the city of Jyväskylä, Finland. Hospitals, physiotherapy clinics and CP associations will be contacted in search of suitable participants. Acquiring the whole sample size in Jyväskylä was not possible, so the recruitment of participants was extended to nearby cities. Furthermore, the coronavirus caused a three-month lockdown in spring 2020, which paused the participation of some participants and delayed recruitment. Twenty-four typically developing age and sex-matched controls will also be recruited from local schools and the University of Jyväskylä. The recruitment has been ongoing since May of 2017 and it is expected to end in December of 2020.

#### Inclusion criteria

The project will accept male and female participants who are aged 9–24 years, with a confirmed diagnosis of unilateral or bilateral spastic CP and are classified as level I to III in the Gross Motor Function Classification System (GMFCS [62];).

#### Exclusion criteria

The project will not accept participants who: a) have had lower limb surgery and/or pharmacological treatments (e.g. intrathecal baclofen, botulinum toxin) in the past six months; b) have had selective dorsal rhizotomy; c) are utilizing serial casting on the lower limbs; d) have participated in a resistance training program for the lower limbs in the last six months; e) are unable to provide sufficient cooperation in the intervention and testing sessions; f) are unable to stand with both heels touching the floor (i.e. ankle in anatomical position). Exceptionally, participants within exclusion criteria a) and b) may be accepted as case studies.

### Sample size

Power calculation was performed for the project’s primary hypothesis (i.e. gait performance), specifically for the effect of the EXECP intervention on walking performance measured by the six minutes walking test (6MWT [63];). The distance covered in the 6MWT is reported to reflect functional capacity and has been recommended as a submaximal exercise test for children with CP of GMFCS I to III [64, 65]. Results from Pre-tests 1 and 2 for the first 9 participants in the project were utilized for sample size calculation utilizing an online spreadsheet (www.sportsci.org [66];). Data was log-transformed due to the inexistence of zero and negative values, yielding a typical error of 1.06, between-subject SD of 1.46 and a smallest important change of 1.08. Utilizing the pre-post parallel-groups RCT model, with a power of 0.8 and an alpha of 0.05, a sample size of 24 participants in both CP and TD group is required. No specific sample size calculation algorithm for our experimental design was found, thus the RCT calculation served as an upper bound for the sample size.

### Study procedures

The EXECP intervention will mainly take place at the NMRC, although it can be performed in other gyms having all required training equipment. It consists of two to three training sessions per week for 12 weeks. The number of weekly sessions will depend on the level of PA of each participant: those engaged in regular weekly PA may choose to perform 2 or 3 sessions, while sedentary participants will train 3 times per week. Training sessions are 90 min in duration and are separated by at least 48 h. Each training session starts with an inclined treadmill walking (5–10 min), followed by strength (60–75 min) and flexibility training (10–20 min). To ensure optimum training quality all sessions are performed individually and supervised by a strength and conditioning coach or a physiotherapist with full understanding of the training protocol.

### Gait training

A portable mechanical treadmill with an inclination of 6° or 7.3° (Vida XL, Venlo, Netherlands) will be utilized for training. Participants are instructed to walk at a comfortable speed, avoiding toe walking and trying their best to attain heel strike. A non-motorized treadmill was chosen because belt movement is attained by pushing it with hip extension and ankle plantarflexion, reinforcing a correct pattern of muscle activation. Verbal feedback will be constantly given during the warm-up to improve gait quality, and the participant will be allowed to stop and rest whenever necessary. In addition to 5–10 min of gait training in every session, all participants will receive a treadmill to take home, and are asked to walk a minimum of 10 min per day every day for the duration of the intervention. An exercise diary will be filled by the participants or their guardians to document the weekly walking durations. During the maintenance period, the participants will choose if they want to keep using the treadmill at home and updating their diary or stop and return it.

### Strength training

Five single joint and five multi-joint exercises were selected for the intervention (Table 1). This composition was chosen because single joint exercises allows greater loading control minimizing compensatory movements [24, 64], whereas multi-joint exercises offer a mixture of strength and motor control training that may be beneficial for motor function. The strength training program has two protocols (A/B), with a minimum of 7 exercises targeting lower limb and trunk muscles. However, depending on the speed that exercises can be performed, which varies considerably between GMFCS levels, 7 to 10 exercises may be performed in every session. The protocols are trained weekly in the order AB (2 sessions per week) or an alternating pattern of ABA/BAB (3 sessions per week).

**Table 1:** Description of the strength training exercises

	Seated Calf Raise	Standing Calf Raise	Seated Dorsiflexion	Seated Machine Knee Extension	Seated Machine Knee Flexion	Seated Horizontal Leg Press	Squat	Hip Flexion	Roman Chair Trunk Extension	Hollow Rock
**Muscles** **targeted**	Soleus, Gastrocnemius	Soleus, Gastrocnemius	Tibialis Anterior	Quadriceps femoris	Hamstrings	Gluteus maximus, quadriceps femoris, hamstrings, triceps surae	Gluteus maximus, quadriceps femoris, hamstrings, triceps surae	Iliopsoas, rectus femoris, sartorius, tensor fasciae latae, tibialis anterior	Erector spinae, multifidus	Trunk flexors, hip flexors, transversus abdominis
**Initial** **Position**	Seated with knees at 90°. Forefoot on a 10 cm step, ankle in maximal attainable dorsiflexion. Weight over the distal thigh of the training leg.	Standing with hips and knees at 0°, and the forefoot on a 10 cm step in maximal attainable dorsiflexion. Holding on parallel bars for balance^a^.	Hips at 70–90°, knees at 0–20°, ankle in full plantarflexion. An elastic band on the forefoot resists the dorsiflexion movement.	Hips at 80°, knees at 115°. Knee and machine’s lever arm center of rotation aligned. Lever arm positioned at the distal shank.	Hips at 80°, knees at 0–5°. Knee and machine’s lever arm center of rotation aligned. Lever arm positioned at the distal shank.	Hips at 110–90°, knees at 80–100°. Feet and knees at hip width. 0–20° of hip external rotation.	Standing with hips and knees at 0°. Holding an adjustable support with both hands. Feet and knees at hip width. 0–20° of hip external rotation.	Supine position, arms laying by the side and both legs touching the mat. An elastic band on the forefoot resists hip flexion and ankle dorsiflexion.	Hips and knees at 0°, the chair is 30–45° inclined. Padded support at pelvis height. Distal posterior part of the shank locked against a padded support.	Supine position, hips and knees at 0°. Arms laying by the side and both legs touching the mat.
**Kinesiologic** **Description**	Unilateral or bilateral ankle plantarflexion.	Unilateral or bilateral ankle plantarflexion	Unilateral or bilateral ankle dorsiflexion.	Unilateral or bilateral knee extension.	Unilateral or bilateral knee flexion.	Unilateral or bilateral hip and knee extension, and ankle plantarflexion.	Bilateral hip and knee extension, and ankle plantarflexion	Unilateral or bilateral hip flexion and isometric ankle dorsiflexion.	Isometric trunk and hip extension.	Isometric trunk and hip flexion. Isometric knee extension.

Hip 0° = anatomical position (positive values = flexion), Knee 0° = fully extended

^a^If the exercise is too hard, leaning on the bars and helping with the arms will be allowed. If the exercise is too easy, it will be done in the leg press machine

Ankle plantarflexors will be trained with seated and standing calf raises. Ankle dorsiflexors will be trained utilizing a rubber band resistance against the dorsiflexion movement. Additionally, TA will also be trained isometrically during a hip flexion exercise, in which the participant laying supine must flex the hip against the rubber band resistance placed on the forefoot. Manual resistance may be used instead of the rubber band in case the trainer perceives it as easier to apply the resistance during the entire ROM. The thigh muscles will be trained utilizing seated knee extensor and knee flexor machines. Additionally, lower limb muscles will be trained using the leg press and squat holding with both hands an adjustable support. A dense foam ball may be placed between the participant’s knees to prevent hip adduction during leg press and squat. Exercises will be done mostly unilaterally due to strength differences between limbs (80–100% of total training volume), except the squat which will mostly be trained bilaterally. Bilateral training may be performed if exercise technique is correct and limb loading appears similar; based on the participant’s perception of fatigue/effort and the trainer’s visual evaluation. Trunk and hip flexors will be trained isometrically with the hollow rocks exercise, in which the participant lies supine on the floor and must lift the legs slightly above the floor (i.e. hip and trunk flexion, knee extension). Trunk extensors will be trained isometrically using an inclined (30–45°) roman chair. Table 1 describes the positioning, target muscles and kinesiology of each exercise.

To enhance autonomy, comfort and interaction within the sessions, participants can choose the order of the exercises if this complies with the general rule of avoiding fatigue build up, which could hinder both execution quality and intensity. More specifically, this rule states that exercises for the same muscle group always must be separated by at least one different exercise (e.g. seated and standing calf raises cannot be executed consecutively). The trainer will guide this process and suggest possible sequences. Table 2 provides an example for both protocols.

**Table 2:** Training progression over 12 weeks (left), and example of exercise list per training session (right)

Week	Volume	Load	Movement Duration (s)	Rest (s)	Session A^a^	Session B^a^
**1–4**	3 sets of 8 repetitions	8 RM	3 concentric 3 eccentric	60	1 – Seated calf raise 2 – Seated dorsiflexion 3 – Standing calf raise 4 – Hip flexion 5 – Seated horizontal leg press 6 – Roman chair trunk extension 7 – Squat	1 – Seated machine knee flexion 2 – Seated machine knee extension 3 – Hip flexion 4 – Standing calf raise 5 – Seated horizontal leg press 6 – Isometric hollow rocks 7 – Squat
**5–8**	3 sets of 8 repetitions	8 RM	1 concentric 3 eccentric	90		
**9–12**	4 sets of 6 repetitions	6 RM	! concentric 2 eccentric	90		

! = ballistic muscle action; *RM* repetition maximum; ^a^ = each session has a minimum of 7 exercises and a maximum of 10 (i.e. all exercises)

The training load was devised to increase muscle strength and mass, complying with the American College of Sports Medicine and National Strength and Conditioning Association guidelines [30, 31] and specific guidelines for CP [25]. The intervention is divided in 3 blocks of 4 weeks: the first block consists of 3 sets of 8 repetitions maximum (i.e. the ninth repetition cannot be attained due to fatigue), movement duration of 6 s (3 s concentric and 3 s eccentric), 60s of rest and no muscle relaxation between repetitions. This training load has been shown adequate to increase muscle strength and mass, and it is very safe as the intensity is approximately 50% of 1 repetition maximum [67]. Furthermore, people with CP usually perform strength exercises with fast concentric bursts and have difficulty in controlling slow eccentric muscle actions. Thus, this protocol also tackles an important motor control deficit in CP. In the second block, the volume is maintained while the intensity is increased by reducing the concentric movement duration to 1 s and increasing the rest to 90 s. In the third block, training volume and rest are maintained, but the sets are increased to four while the repetitions are decreased to 6, concentric muscle actions are now done as fast as possible while eccentric muscle action duration is reduced to 2 s. Thus, concentric power is trained, and the modifications permits further increases in exercise intensity. Table 2 depicts the training load progression during the intervention.

Exceptionally, the squat exercise will follow a different progression because of its inherent higher intensity (due to body weight): one to four sets of 10 repetitions with the largest attainable ROM will be performed. Movement duration is similar to the other exercises, while the rest starts at 90 s, and will be decreased when possible to 60 s. After the entire volume is executable with 60 s of rest, balance disks (Casall, Vantaa, Finland) will be placed under the participant’s feet to cause instability and increase exercise difficulty, also unilateral squats may be utilized to further increase the exercise intensity.

An important aspect of isoinertial strength training is that the ability to produce torque varies throughout the joint ROM, due to the sarcomere force-length curve and the joint’s lever arm [68]. Furthermore, CP joint ROM is often reduced [69] and muscle weakness is present [24]. Thus, allowing CP participants to perform the strength exercises by themselves leads to a movement with a smaller joint ROM (as compared to the total possible active ROM), and the non-optimum part of the torque-angle curve is not trained. Since strength gains are generally higher at the trained angles (i.e. length-specific adaptations [70, 71];) and increasing the torque production in the whole active curve seems very reasonable to allow functional benefits, an assisted training procedure will be adopted. The trainer will actively help the participant on the concentric phase of the movement in the positions in which the participant is not able to perform by himself. The exercise resistance will be selected based on the participant’s strength on the optimal angles, thus the trainer will always be assisting on non-optimal angles, and never increasing the resistance. The eccentric phase will be performed unassisted, but constant feedback about movement velocity will be given. Due to the assistance during every repetition, no 8 or 6 repetition maximum test will be performed to adjust the weight, rather the trainer will frequently ask the participant to try and perform one more repetition, if successful, the weight will be increased for the next set. Whenever a participant is unable to perform a minimum concentric ROM, the protocol will be adjusted to include an isometric muscle action of 3 s in each repetition. Thus, it will be a small concentric movement, followed by the isometric hold phase and then the eccentric phase. If during the intervention the concentric movement increases, the isometric part will be removed. At the start of each training session, the trainer will ask the participant if any adverse events were experienced after the previous session. Participants will be constantly reminded to provide immediate feedback about any pain or discomfort during the training sessions.

### Flexibility training

Four sets of 45 s passive-static stretching at the pain threshold (i.e. position in which the participant acknowledges an initial stretch pain sensation) will be performed for each muscle group diagnosed short in the pre-tests. One and two-joint hip flexors will be stretched in the Modified Thomas Test position [72]. The participant lies supine on a table holding one of the lower limbs in full hip flexion (assistance may be provided), while the other leg hangs outside the table (i.e. hip extension). The only difference between the stretches is that the trainer will apply the hip extension torque at the distal thigh with the knee joint positioned either in full flexion (two-joint stretch) or in a relaxed position (one-joint stretch). The seated butterfly stretch will be utilized to stretch the hip adductors. The participant will have his back supported on a wall, hips externally rotated, knees flexed and the soles of the feet in contact. The trainer will use his knees to keep the participant’s feet in position while pressing down the thighs causing hip abduction. Hamstring stretch will be performed in supine position, the trainer will secure the untrained leg flat against the floor, then flexes the participant’s hip approximately to 90° and applies a knee extension torque at the posterior aspect of the shank. No flexibility training will be performed for the triceps surae muscle group because of the following reasons: 1) lack of feasibility to execute the selected stretching protocol bilaterally for all tested muscles (session duration would greatly exceed 90 min); 2) the inclined gait training forces the ankle joint into dorsiflexion during the stance phase, working as a passive dynamic stretch. To check if the gait intervention by itself can change flexibility, it seemed reasonable to avoid stretching triceps surae and to focus on the other muscle groups.

### Testing sessions

Participants will attend the NMRC and the Centre for Interdisciplinary Brain Research (CIBR) at the University of Jyväskylä for all testing sessions. Each testing point (pre-test 1 and 2, post-test 1) consist of three testing sessions (S1, S2 and S3) performed 4 h to seven days apart. Exceptionally, post-test 2 is composed only of S1 and a modified S2. Session 1 (S1) consists of the following tests: joint flexibility, electrostimulation (H-reflex and PAD), muscle strength and tonic stretch reflex threshold. Session 2 (S2) consists of the following tests: blood sampling, body composition and height, arterial stiffness, instrumented gait analysis, six minutes walking test. In session 3 (S3) magnetoencephalography measurements are performed. The modified S2 performed in post-test 2 consists only of the gait tests and height measurement. Table 3 describes the timeline of each testing session. The TD group will only perform the pre-tests 1 and 2. A familiarization session will be performed before the pre-test 1 to get the participants acquainted with the tests. Additionally, this session will be utilized to duplicate some tests of S1 and S2 for a repeatability study.

**Table 3:** Testing sessions timetable and procedures

Testing session 1 (S1)	Testing session 2 (S2)	Testing session 3 (S3)
08.00 – Flexibility tests (Hip and Knee)^b^;	08.00 – Blood sampling;	08.00 – Participant preparation;
08.15 – H-reflex/PAD protocol for Sol^a^ at rest;	08.10 – Body composition, height and arterial stiffness;	08.45 – Resting-state;
08.40 – Flexibility test (ankle)^b^;	08.30 – Breakfast;	09.05 – Proprioceptive stimulation for TS^a^;
08.50 – Preparatory activity PF^a^/DF^a^;	09.00 – Participant preparation;	09.30 – Preparatory activity DF^a^;
09.00 – MVC (PF^a^/DF^a^);	09.50 – Gait preparatory activity;	09.40 – Isometric DF MVC^a^;
09.20 – TSRT protocol (TS^a^);	09.55 – 6 × 1 min gait test;	09.50 – Cortico-muscular coherence protocol^a^;
09.30 – Preparatory activity KE^a^/KF^a^;	10.10 – Rest;	10.01 – Session ends.
09.40 – MVC (KE^a^/KF^a^)	10.25 – 6 min walking test;	
10.00 – Session ends.	10.31 – Session ends.	

*NMRC* Neuromuscular Research Center, *CIBR* Centre for Interdisciplinary Brain Research, *PAD* post-activation depression, *Sol* soleus muscle, *MVC* maximum voluntary contraction, *PF* plantarflexion, *DF* dorsiflexion, *TSRT* tonic stretch reflex threshold, *TS* triceps surae muscle, *KE* knee extension, *KF* knee flexion; ^a^most affected limb is tested; ^b^ both limbs are tested

### Primary outcome measure

The six minutes walking test will be performed on an indoor 30 m rubber track, and its primary result is the distance that the participant is able to walk in 6 min. Heart rate (Polar Electro Oy, Kempele, Finland) will be measured to quantify exercise intensity (i.e. mean, maximum and minimum). An inertial measurement unit (NGIMU, X-IO Technologies, UK) will be firmly strapped at the participant’s lower back slightly above the posterior superior iliac spine. Gait variability will be calculated with refined composite multiscale entropy [73, 74] and refined multiscale permutation entropy [75, 76]. Three-dimensional (3D) raw acceleration signals and the resultant acceleration will be utilized for this calculation. Following the approach by Ihlen et al. [77], entropy will be calculated with coarseness scales of τ = 1 to 20, the length of the template for entropy evaluation equals four and the tolerance for refined composite multiscale entropy will be set at 0.3 times the standard deviation of the entire test. Raw signals will be sampled at 100 Hz, and stored on a SD card for later offline analysis utilizing a freely available java implementation (https://github.com/tjrantal/javaMSE). Cross-sample entropy [78, 79] will be utilized to compare the pre and post-tests for each participant.

### Secondary outcome measures

#### Gait analysis (kinematics, kinetics, gait variability)

3D lower limb kinematics will be captured utilizing a Vicon system (Vicon Motion Systems, Oxford, UK), consisting of 8 cameras (MXT40) with a sampling frequency of 200 Hz. Reflective markers will be placed at: (1) medial and lateral malleolus; (2) second metatarsal head; (3) heel; (4) lateral aspect of the shank; (5) medial and lateral femoral epicondyles; (6) three non-collinear markers at the lateral aspect of the thigh; (7) anterior superior iliac spine; (8) posterior superior iliac spine. This marker set was chosen to allow calculation of both direct kinematics with the Vicon Nexus 2.5 lower limb gait plug-in [80, 81] and inverse kinematics with the free open-source OpenSim software (http://opensim.stanford.edu/; [82]). Ground reaction forces will be simultaneously acquired at 1 kHz using two force plates (51 cm × 46 cm, AMTI OR6-6-2000, Watertown, USA) on a 7.4 m × 0.6 m rubber walking path. Inverse dynamics will be utilized to calculate joint moments using both OpenSim and Vicon Nexus for comparison purposes. Gait variability will be calculated utilizing the same procedures previously explained in the 6MWT. Gait cycles will be identified using footswitches (Noraxon, Scottsdale, USA) with two sensors placed bilaterally on the heel and forefoot.

The test consists of 6 trials of 1 min, in which participants must walk back and forth at a maximum safe and controlled walking speed. Participants are instructed to disregard the force plates (i.e. not modify the gait to step on them) and to inform in case they are feeling tired. A preparatory activity of 2 min with progressively higher walking speeds will be performed before the test. Rest periods of 1–2 min between trials based on the participant’s feedback will be given. Each trial can be stopped at any time for adjustments or resting, nonetheless a total of 6 min of walking will be recorded.

#### Muscle strength

Maximal isometric and concentric ankle plantarflexion and dorsiflexion will be assessed using a custom-build motor driven dynamometer (NMRC, University of Jyväskylä, Finland). Participants will be seated with the knee joint fully extended, hip joint flexed at 60° (anatomical position = 0°), and the ankle joint at an initial position of 0° (i.e. the sole of the foot at right angles to the tibial axis) or 28° into plantarflexion. The foot will be firmly attached to a footplate mounted on the rotation platform so that the rotation axes of the ankle joint and the motor-driven platform coincide. Participants will be securely stabilized by an assembly of straps that fastened both shoulders and connected to a waist belt. An additional strap with a foam support prevents the knee joint of the tested leg from flexing. The torque around the rotational axis of the motor will be measured by a piezoelectric crystal transducer (Kistler Holding, Winterthur, Switzerland), and the ankle joint angle will be monitored by a linear potentiometer. Furthermore, a small stiff metal wire attached to a spring system, located under the calcaneus, will continuously monitor heel displacement from the footplate. Torque, joint angle, and heel displacement signals will be sampled at 1 kHz with a 16-bit A/D converter (CED power 1401, Cambridge Electronics Design, Cambridge, UK). The plantarflexion test starts with a 2 s maximal isometric muscle action at 0°, followed by an isokinetic (14°/s) concentric effort until 28°. The dorsiflexion test starts with a 2 s maximal isometric muscle action at 28°, followed by an isokinetic (14°/s) concentric effort until 0°. The highest attained torque at 0°, 5.5°, 11°, 16.5°, 22°, and 27.5° among the trials will be used for analysis. This approach will be used because force fluctuations during the trials (i.e. short moments of relaxation) often happen when testing the CP group, thus it may be that one single trial will not have all the peak torque values for all joint angles.

Maximal isometric and concentric knee flexion and extension will be assessed using a custom-build motor driven dynamometer (NMRC, University of Jyväskylä, Finland). Participants will be seated with the knee joint fully extended (0°) or at 75° of flexion and hip joint flexed at 80°. The distal part of the shank will be secured with a velcro strap to a strain gauge (NMRC, University of Jyväskylä, Finland) capable of measuring both tensile and compressive forces. The position of the strain gauge on the lever arm is adjustable and the distance from the axis of rotation will be individually recorded and reproduced for all following measurements. The rotation axis of both dynamometer and knee will be carefully aligned during a submaximal voluntary muscle action, to take into account the system’s compliance. Participants will be securely stabilized by an assembly of straps fastening both shoulders and connected to a waist belt. The knee flexion test starts with a 2 s maximal isometric muscle action at 0°, followed by an isokinetic (15°/s) concentric effort until 75°. An examiner will keep strong downward pressure at the distal part of thigh to prevent hip flexion during the trial. The knee extension test starts with a 2 s maximal isometric muscle action at 75°, followed by an isokinetic (15°/s) concentric effort until 0°. Torque will be obtained by multiplying the moment arm by the force, and joint angle will be monitored by a linear potentiometer. Both signals will be sampled at 1 KHz with the same hardware and software utilized for the ankle torque tests. The highest attained torque in non-overlapping 5° steps among all trials will be used for analysis. For all four strength tests, rate of force development will be calculated as the maximum slope during the 0–200 ms time period in the isometric part. All measurements will be performed on the most affected leg for the CP group, and on the corresponding leg on the matching control participant. Test isokinetic velocity was chosen based on the slowest intervention training velocity (3 s muscle action for the ankle joint with a ROM of ~ 45°), and because slower velocities are easier to be well performed by the CP participants (observed in the pilot experiment). A preparatory activity consisting of ten progressively stronger efforts from 20 to 90% of the maximum voluntary contraction (MVC) will be performed before each test. Visual torque feedback will be provided in real-time and participants will receive strong verbal encouragement during every trial. Three to five trials with 1–2 min of rest for each test will be performed. In addition to the specific torque-angle analysis described, joint angles in which torque is greater than 90% of the peak concentric torque (i.e. optimum angle; [83]) and greater than 50% of the peak concentric torque (i.e. curve width; [84]) will also be calculated.

#### Electromyography

Three EMG setups will be utilized in the project. The first setup will be used for the strength, ankle flexibility and neural tests (i.e. H-reflex, PAD and tonic stretch reflex threshold). EMG activity will be recorded from Sol, MG and TA muscles with self-adhesive electrodes (Blue Sensor N, Ag/AgCl, 0.28 cm^2^; Ambu, Ballerup, Denmark), and a ground electrode placed on the tibia. Electrode placement and skin preparation will be performed according to SENIAM [85]. The electrodes will be placed on the muscle belly in accordance with the underlying fiber direction (20-mm-interelectrode distance). EMG signals will be amplified and high-pass filtered (1000X, 10 Hz) by a preamplifier (NL824; Digitimer, Welwyn Garden City, UK) and then band-pass filtered (10 Hz to 1 kHz) by a differential amplifier (NL900D/NL820A; Digitimer Ltd., UK). The signals will be acquired on a personal computer at a rate of 5 kHz via a 16-bit A/D converter (CED power 1401; Cambridge Electronics Design, Cambridge, UK). EMG during the maximal voluntary plantarflexion and dorsiflexion trials will be quantified in 200 ms root mean square (RMS) windows preceding the joint angles of 0°, 5.5°, 11°, 16.5°, 22° and 27.5°. TA coactivation during plantarflexion will be expressed as % of the maximal RMS EMG obtained on the dorsiflexion trial at the same joint position. The same procedure will be performed to express Sol and MG coactivation during ankle dorsiflexion.

The second setup utilizes a wireless EMG system (Noraxon, Scottsdale, USA) that will acquire EMG activity during the instrumented gait analysis. Data acquisition configuration, muscles tested, and electrode placement are identical to setup 1, except for two differences: 1) sampling frequency is 1.5 kHz; 2) two sets of bipolar electrodes will be placed in TA muscle, 8–10 cm apart depending on the subject’s tibia length.

The third EMG setup is built-in the MEG System (Elekta Neuromag TRIUX, Elekta Oy, Helsinki, Finland). The same bipolar electrode placement as in setup 2 will be utilized, with a MEG compatible electrode (Neuroline 720, Ag/AgCl, 0.28 cm2; Ambu, Ballerup, Denmark). EMG signal will be sampled at 1 KHz and band-pass filtered 1–1000 Hz.

#### Neural parameters

H-reflexes and M-waves will be evoked in Sol by percutaneous electrical stimulation of the tibial nerve, while the participant lies in prone position. A single rectangular pulse (1 ms) will be delivered from a constant-current stimulator (DS7AH; Digitimer, UK). A circular cathode with a pickup area of 0.77 cm^2^ (Unilect4535M, Ag/AgCl; Unomedical, Redditch, UK) will be placed over the tibial nerve on the popliteal fossa, and an oval-shaped, 5.1 × 10.2 cm anode (V-trodes; Mettler Electronics, Anaheim, CA) will be placed above the patella. The stimulation site providing the greatest amplitude of evoked responses in Sol will first be located by a handheld cathode electrode that will be later replaced by a self-adhesive electrode. The full recruitment curve will be attained by starting with a stimulation intensity of 3 mA and increasing in 0.5–2 mA steps (i.e. smaller steps closer to the maximum H-reflex) at 0.1 Hz until the Maximal M-wave is reached. Peak-to-peak values for maximum H-reflex and M-wave will be computed.

The PAD protocol will be performed after the recruitment curve with the participant in the same position. Stimulation intensity will be first adjusted to evoke H-reflex responses corresponding to 75% of maximum H-reflex at 0.1 Hz, then sixteen stimuli at two frequencies (0.1 and 1 Hz) will be performed. H-reflexes will be normalized by the preceding M-wave, and PAD will be computed as the ratio between 1 Hz and 0.1 Hz (i.e. greater values means less PAD). This normalization procedure is important because the H-reflex is directly influenced by the preceding M-wave (i.e. effective stimulation intensity). However, since the stimulation intensity is constant and supposedly so is the preceding M-wave, previous studies [86, 87] have chosen to report the ratio between the absolute peak-to-peak H-reflex values. Thus, for comparison purposes the absolute PAD ratio will also be calculated.

Stretch hyperreflexia of Sol and MG will be assessed utilizing the TSRT [88, 89]. The participant will be seated with the knee joint fully extended and the ankle dynamometer previously described will induce stretches (from 20° of plantarflexion to 0°) on triceps surae at four different velocities: 50, 100, 200 and 300°/s. The stretch velocities will be delivered in a pseudo-randomized and balanced order, yielding 10 stretches in each velocity, with an interval of 2.6–2.9 s. EMG data will be used to determine the joint angle in which the stretch reflex onset occurred (i.e. dynamic stretch reflex threshold, DSRT). A regression line based on a first-order linear equation using all DSRT will be calculated on a stretch velocity-joint angle plot. From the equation, the coefficient of determination, slope and intercept with the x-axis will be attained. The TSRT is the angle at which the regression line intersects with the x-axis, larger values indicate a high level of stretch hyperreflexia. TSRT will be calculated separately for each muscle. In addition to the already established TSRT calculation, we propose using the latency of the H-reflex to take into account the time between the stretch reflex onset and the EMG burst onset. A typical H-reflex latency of 35 ms and a stretch velocity of 300°/s can cause a difference of 10.5° between the stretch reflex and EMG onset, provoking a systematic error towards higher velocities. Thus, all variables in the TSRT will be calculated with and without the latency correction, and the differences will be discussed. Participants will be instructed to relax and will be wearing noise blocking headphones. Trials with EMG exceeding 5% of the isometric plantarflexion MVC in the 500 ms preceding the stretch will be discarded. The EMG burst onset will be defined as a 3SD increase in the RMS of a 20 ms moving average for at least 20 ms compared to the pre-stretch state. Visual inspection of false positives due to small brief muscle activations will be performed and automated onset detection algorithms may also be utilized. H-reflex, PAD and TSRT will be assessed on the most affected limb of the CP group and in the same leg of the age/sex matched control.

Frequency domain analysis of the EMG will be performed in the following conditions: A) intramuscular TA coherence during the swing phase of gait; B) cortico-muscular and intramuscular TA coherence during a sustained isometric ankle dorsiflexion. Condition A will be collected with EMG setup 2, during the instrumented gait analysis test (S2), and B will be collected in the MEG session (S3), using EMG setup 3. Surface EMG will be preprocessed using full-wave rectification as it has been shown to be appropriate for low force muscle actions [90–92]. The finite Fourier Transform using the method of disjoint sections will be utilized [93], in which the complete EMG record R (i.e. approximately 60 s for A, and 480 s for B) is divided in L non-overlapping disjoint sections of length T, where R = LT. For A, T will be selected to reach a spectral resolution ≤5 Hz. If possible, the swing phase will be divided into two or three parts, depending on the actual length of these phases and the achieved frequency resolution. For B, R = 480 s and T = 1 s, yielding a frequency resolution of 1 Hz. The coherence between two rectified EMG signals (x and y) is defined as their cross-spectrum normalized by the auto-spectra for a given frequency (λ), according to the following equation:

$$ {\left|{R}_{xy}\left(\lambda \right)\right|}^2=\frac{{\left|{f}_{xy}\left(\lambda \right)\right|}^2}{f_{xx}\left(\lambda \right){f}_{yy}\left(\lambda \right)} $$

Coherence measures how closely two signals are related by a linear transformation [93, 94], providing a bounded and normative measure of association, taking values between 0 and 1. A perfect linear relationship has a value of 1, while full independence has a value of 0. Pooled coherence estimates will be calculated to characterize both control and experimental groups [95]. The estimation of pooled coherence joins separate signals from different subjects into a single longer signal, and then calculates the coherence of this record using the periodogram approach. Pooling the EMG signal of different participants may result in a non-constant standard deviation (i.e. nonstationarity) between the different sections, in this case signals will be normalized to have unit variance [96].

#### Joint flexibility and triceps surae mechanics

Lower limb joint flexibility will be assessed using three tests: the modified Thomas test, the passive knee extension test and a passive ankle dorsiflexion test. In the modified Thomas test, three measurements are performed at the hip joint and 2 at the knee joint [72]. The first hip measure has the goniometer fulcrum at the greater trochanter of femur, reference lines using lateral femoral condyle and a line perpendicular to the bench. The second hip measurement is like the first, however the researcher will apply a hip extension torque at the distal thigh and will record the angle at which the subject reports initial stretch pain sensation. The third hip measure has the goniometer fulcrum at the anterior superior iliac spine, references lines using the superior aspect of the patella and a line parallel to the bench. The first knee measure has the goniometer fulcrum at the lateral femoral condyle, reference lines using lateral malleolus and greater trochanter of femur. A limitation of the knee angle to access two-joint hip flexors flexibility is that it is directly affected by the hip angle 1 (one-joint hip flexors). Thus, the second knee angle is measured with the participant in the same position, except that the thigh of the tested leg is supported on the bench (instead of hanging outside of it), thus hip angle will be 0°. It should be noted that only hip measurement 2 is a flexibility test, because attaining maximum ROM requires unlimited torque and the subject acknowledging stretch sensation pain. On the other four measurements, the joint angle attained is a result of the interaction between gravity and the joint’s passive resistance to stretch. Thus, a stiffer joint will not yield much to the gravity force, and the reduced ROM cannot be mistakenly interpreted as poor flexibility. Although not true flexibility tests, these measurements provide important information about how different muscles affects the lower limb joints.

The passive knee extension test is performed in the same position as the hamstring stretch with the participant lying supine with hip and knees at 90°. The non-tested thigh is strapped to the bench to avoid pelvic retroversion during the test. The examiner slowly applies torque at the posterior aspect of the shank causing knee extension, while another examiner maintains the hip position. The test stops when the participant acknowledges an initial stretch pain sensation. A similar knee angle measurement used in the modified Thomas test is performed. A manual goniometer is utilized to perform the both tests.

The passive dorsiflexion test will be performed using the same motor-driven dynamometer utilized for the ankle strength tests. The participant will seat with the hip joint flexed at 60°, ankle at 35.5° of plantarflexion and knee on two possible positions: 90° or 0°. The examiner will slowly (< 5°/s) move the device causing ankle dorsiflexion until the participant acknowledges an initial stretch pain sensation. Another criterion to stop the test is a heel raise higher than 5 mm. Since the motor-driven dynamometer will be also utilized subsequently to perform very fast dorsiflexions for the TSRT test, for safety reasons the maximum attained dorsiflexion angle is 21°. Thus, it is possible that maximal ROM is not measurable for some participants in the extended knee position. Three to five trials in each knee position will be performed and the maximum ROM for each position and leg will be reported. From the passive dorsiflexion torque-angle curve, in the region encompassing 20–80% of the participant’s peak torque [51], average joint stiffness (i.e. first derivative), energy (i.e. integral) and hysteresis (difference between loading and unloading integral) will be calculated. Additionally, these variables will be calculated utilizing absolute joint angles. Trials with triceps surae EMG RMS higher than 3 SD of resting levels will be discarded (EMG setup 1). Table 4 presents the criteria for each joint flexibility measurement based on TD normative data [97–103].

**Table 4:** Joint flexibility tests and references from typically developing controls attained in other studies

Reference Values and Diagnostic	
**Modified Thomas test**	
Hip measurement 1: Hip angle > 0° = short and/or stiff one-joint hip flexors (i.e. iliopsoas, hip adductors).	
Hip measurement 2 (examiner applies hip extension torque): Hip angle > 0° = short one-joint hip flexors.	
Hip measurement 3: Reference value = 0° (i.e. ASIS and patella aligned on the sagittal plane). Positive values = short and/or stiff hip abductors.	
Knee measurement 1: Knee angle < 67° = short and/or stiff two-joint hip flexors (i.e. rectus femoris, tensor fascia latae, sartorius).	
Knee measurement 2 (supported hip): Knee angle < 67° = short and/or stiff two-joint hip flexors.	
Note: Tightness of the anterior capsule and ligaments may also influence the test.	
References: [72, 97–99]	
**Passive knee extension test**	
Knee angle > 40° (with the hip at 90°) = short hamstring muscles.	
References: [100, 101]	
**Passive ankle dorsiflexion test**	
Ankle angle < 18° with knee at 0° and 90° = short Soleus muscle.	
Ankle angle < 18° with knee at 0° and ankle angle > 18° with knee at 90° = short Gastrocnemius muscle.	
Ankle angle **>** 18° in with knee in 0° and 90° = good triceps surae flexibility.	
References: [102, 103].	

*ASIS* anterior superior iliac spine. Hip measurements 1–2: 0° = anatomical position, positive values = hip flexion. Knee measurements: 0° = knee in full extension. Ankle measurements: 0° = sole of the foot at right angles to the tibial axis, positive values = dorsiflexion

Neuromechanical responses during the TSRT protocol (i.e. 40 stretches at 4 velocities) will also be analyzed. The higher stretch velocities in this protocol will result in reflex muscle activation, thus involving both passive and active elements. First, mechanical artifacts at the beginning and end of each stretch will be removed, then peak and mean torque, joint stiffness and energy will be calculated for each stretch. Additionally, maximum EMG RMS (EMG setup 1) will be calculated with a 50 ms moving window for each stretch. Comparisons between velocities and between the first and last group of 8 stretches will be performed to gain insight into the differences in neuromechanical responses to dynamic repetitive stretching.

#### Physical activity

PA will be assessed using questionnaires and device-based measurements. The questionnaire from Jackson et al. [104] with a scale from 0 to 7 describing the overall PA level is complemented by questions about the amount of moderate-to-vigorous PA during a week. PA will also be measured with a tri-axial waist-worn accelerometer (X6-1a, Gulf Coast Data Concepts Inc., Waveland, MS, USA) with a dynamic range of ±6 g and a sampling frequency of 40 Hz. The accelerometer will be utilized during seven typical days at three time points: control, intervention and maintenance periods. A minimum of 3 days with 8 h of measurement will be accepted for analysis as it has been shown to provide adequate reliability for children and adolescents with CP GMFCS I [105]. Raw acceleration data will be transformed to Actigraph counts [106], in order to utilize validated GMFCS specific thresholds for different intensity zones for children with CP [107].

Specialized shorts with textile electrodes (Myontec Ltd., Kuopio, Finland, 108) will be utilized to record EMG bilaterally from quadriceps and hamstring muscles during an entire typical day. A small recording electronic module is able to collect muscle activity data for 8–12 h and can be analyzed offline afterwards. EMG signals will be acquired with a sampling frequency of 1 KHz and band-pass filtered (50–200 Hz, − 3 dB). The raw signals will be full wave rectified and averaged over 100 ms non-overlapping windows and stored into a portable module. Daily muscle inactivity, light, moderate and vigorous activity time of the participant will be extracted [108, 109]. Muscle inactivity will be defined as < 90% of the standing EMG activity for each muscle group. Moderate activity threshold is the preferred walking EMG activity, and vigorous activity is twice the amplitude of walking [110]. The participants will receive a diary to record times when the accelerometer and EMG pants are worn, facilitating the removal of non-wear time in the analysis.

#### Gross motor function

Gross motor function will be assessed in the CP group using the 66-item version of the Gross Motor Function Measure (GMFM-66; [111]). GMFM-66 is a valid, reliable and responsive observational instrument, based on interval scaling to evaluate changes in gross motor function in participants with CP [19]. Dimensions D (standing) and E (walking, running and jumping) will be utilized. The test will be video recorded and scored subsequently by the same evaluator. Absolute and relative (i.e. percentage of maximum) scores for each dimension will be reported.

#### Biological maturity

To control for the confounding effect of maturation on the dependent variables during the study, estimated time from peak height velocity (i.e. maturity offset) will be computed using the sex-specific equation provided by Moore et al. [112]:

$$ \mathrm{Girl}'\mathrm{s}\ \mathrm{maturity}\ \mathrm{offset}=-7.709133+\left(0.0042232\ast \left(\mathrm{age}\ast \mathrm{height}\right)\right);{\mathrm{R}}^2=0.898;\mathrm{SEE}=0.528; $$

$$ \mathrm{Boy}'\mathrm{s}\ \mathrm{maturity}\ \mathrm{offset}=-7.999994+\Big(0.0036124\ \mathrm{x}\ \left(\mathrm{age}\ \mathrm{x}\ \mathrm{height}\right),{\mathrm{R}}^2=0.896;\mathrm{SEE}=0.542. $$

#### Body composition, height and cardiometabolic risk factors

Body height will be measured with the head positioned in the Frankfurt plane with a wall-mounted stadiometer and body composition with a bioelectrical impedance device (InBody 720, Seoul, South Korea; [113]). Venous blood samples will be drawn after standard overnight (12 h) fast. Serum insulin will be measured by electrochemiluminescence immunoassay (Immulite 2000, Siemens, Germany). Fasting plasma glucose, total plasma cholesterol, plasma high and low-density cholesterol and plasma triglycerides will be measured by enzymatic colorimetric assays (Konelab 20XTi, Thermo, USA). Insulin sensitivity estimated by the Homeostatic Model Assessment for Insulin Resistance (HOMA-IR) will be calculated using the formula: fasting serum insulin x fasting plasma glucose/22.5 [114]. White and red blood cells, hematocrit and hemoglobin will be measured by an automated analyzer (Sysmex XP300, Sysmex, Japan). Systolic and diastolic blood pressure, aortic pulse wave velocity, and augmentation index will be measured in supine position with a non-invasive oscillometric device after 10 min of rest (Arteriograph, Tensiomed Ltd., Budapest, Hungary; [115]). Participants will be allowed to drink water and take their usual medications during fastening.

#### Magnetoencephalography (MEG)

Participants will be seated inside a magnetically shielded room (VACOSHIELD, Vacuumschmelze GmbH & Co. KG, Hanau, Germany) with their heads positioned within the sensor array of the whole-head-MEG System (Elekta Neuromag TRIUX, Elekta Oy, Helsinki, Finland), consisting of 204 planar gradiometers and 102 magnetometers. MEG data will be acquired at 1000 Hz and band-pass filtered (0.1–330 Hz).

##### Subject preparation

Five head position indicator (HPI) coils will be attached to the participant’s scalp to measure and follow head position with respect to the MEG sensors. Positions of the HPI coils, three anatomical landmarks (nasion, left and right preauricular points), and ~ 150 additional points on the head surface will be determined with a Fastrak 3D digitizer (Polhemus, Vermont, USA). Vertical and horizontal electro-oculograms will be recorded with two pairs of electrodes. The first pair is placed above and below the right eye and the second pair lateral to the outer canthi of the eyes. Electrocardiography will be recorded with a pair of electrodes placed 3–5 cm bellow right and left clavicle and with 8–12 cm inter-electrode distance. The ground electrode will be placed on the right clavicle. EMG activity will be recorded from Sol, MG and TA muscles (EMG setup 3).

##### Equipment

Two custom-made MEG-compatible devices (University of Jyväskylä, Jyväskylä, Finland) were utilized in the study: a pneumatic ankle-movement actuator and a dynamometer capable of measuring isometric ankle plantarflexion and dorsiflexion forces. The chair was modified by removing its shank and foot support and installing a metallic rail (aluminum and brass) to allow adjustable attachment of both equipment to it.

The pneumatic ankle-movement actuator was adapted from the device made by Piitulainen et al. [116]. The operating principle of the MEG compatible pneumatic actuators is reported in Piitulainen et al. [117]. In brief, the actuator is composed of four pneumatic muscles (DMSP-10-120 N-AM-CM, Festo, Vantaa, Finland) embedded into a plastic and aluminum frame with a rotating platform that supports the participant’s foot. The rotation occurs in the sagittal plane (i.e. ankle dorsiflexion) when the internal pressure of the pneumatic muscles is increased (from 1 to 7 bar) and returns to the initial position when the air pressure is released. Rotation axes of the actuator and ankle joint can be aligned. The foot is fixed to the rotating platform by a strap passing firmly around the forefoot.

The isometric dynamometer composed of a metallic plate in L format, with a hard foam support for the posterior distal shank and for the heel. An adjustable metal attachment is positioned on the person’s forefoot. The shank-support is embedded with a load cell and has a strap to fasten the shank, enabling it to measure isometric knee extension and flexion forces. Another load cell is embedded in a support part holding the adjustable metal attachment. A strap firmly secures the participant’s forefoot and thus the load cell can measure both isometric ankle plantarflexion and dorsiflexion forces.

##### MEG protocol

Before the participant enters the shielded room, a 5-min empty room measurement will be recorded to estimate intrinsic noise levels of the MEG sensors for the needs of later data analysis. Three experimental MEG tasks will be performed in the following order: (1) resting state, (2) proprioceptive stimulation and (3) isometric muscle action. The resting-state task consists of two 5-min recordings, in which the participant will remain relaxed fixating on a cross on a screen positioned 1 m away from the participant’s eyes. In the proprioceptive stimulation, the pneumatic ankle-movement actuator will evoke a total of 100 dorsiflexions (movement range from 0° to ~ 15°) with a moderate velocity (mean: 15°/s; peak: 35°/s) every 4 s with ±0.2 s random jitter between the consecutive stimuli. Participants will be instructed to relax and not to resist the movement, fixate on the fixation cross, and avoid excessive eye and head movements. The view of the foot and actuator is blocked, and Brownian noise will be played through earplugs in an individually adjusted volume to mask the noise generated by the movement actuator. In the third task, first a preparatory activity of ten 2-s isometric dorsiflexions with increasing intensity (i.e. ~ 20 to 90% of MVC) at 0° ankle angle in the ankle dynamometer will be performed. Then, in the same position, three to five maximum isometric dorsiflexions (~ 3 s each) will be performed, followed by four sets of submaximal isometric dorsiflexions at 10% of MVC with 120 s of duration each. Visual torque feedback will be provided in real-time, and participants will be instructed to maintain as stable as possible torque throughout each submaximal trial. To avoid fatigue, 1–2 min of rest periods will be enforced between trials (except in the preparatory activity). If the participant is unable to perform dorsiflexion adequately, plantarflexion will be used. Continuous HPI tracking will be performed for later correction of head movements.

##### MEG data preprocessing

First, visual data inspection will be performed to identify bad channels and periods with intense noise. Maxfilter (ver. 3.0, Elekta Neuromag Oy, Helsinki, Finland) with spatiotemporal signal space separation method [118] will be used to reduce endogenous and exogenous sources of noise in the MEG signals. Additionally, MaxFilter will be utilized for head movement compensation during MEG recording and to align the head coordinates across the different longitudinal recording sessions (pre-tests 1 and 2, and post-test 1). The coordinate transformation will be done to the participant individual mean reference head position within each MEG task separately. Artifacts caused by eye movements and cardiac activity will be identified and removed using the electro-oculograms and electrocardiography signals in combination with independent component analysis implemented in MNE-Python software package [119, 120].

##### Data analyses

All further analyses of the MEG data will be processed and analyzed with the MNE-Python software package. The data will be averaged across proprioceptive stimuli in order to examine the passive-movement-evoked fields, and the respective modulation of the rhythmic activity in primary sensorimotor cortex at alpha, beta and possibly also at gamma frequency bands. Cortico-muscular and intramuscular coherence for TA will be computed as described in the EMG section. Resting state data will be analyzed with a special focus on the sensorimotor cortical network. Torque steadiness during the 8 min of submaximal effort will be calculated utilizing the standard deviation and coefficient of variation.

### Statistical analyses

Mean/median values, standard deviations and 95% confidence intervals will be reported. Normality of data will be tested using the Shapiro-Wilk test. General linear mixed model analysis will be utilized to study the differences between time (2 pre-tests and 1 or 2 post-tests) in the CP group. Independent t-tests will be used to compare groups (i.e. CP vs. TD) and paired t-tests to compare the two TD testing times (pre-test 1 and 2). Pearson correlation analysis will be performed to check association between dependent variables. The interference of variables such as PA levels or maturity offset will be controlled if they have a strong linear relationship with other dependent variables. Nonparametric tests may be utilized if necessary. Significance level will be set at *P* < 0.05.

## Discussion

The current training intervention protocol combines different training modalities in an attempt to enhance motor function in people with CP. The individually tailored aspects are: 1) flexibility training for muscles diagnosed short in the Pre-tests; 2) the number of training sessions and strength exercises in each session will be adjusted depending, e.g., on the participants’ motivation, stamina and schedule; 3) introduction of a short isometric hold (3 s) in case that the joint range of motion is limited; 4) stabilization procedures to ensure proper execution of the movements, e.g., using a foam ball to prevent hip adduction during leg press.

The major limitation in this study is the effect of the growth spurt on the dependent variables. If it happens during the control period, results between pre-test 1 and 2 may be considerably different, making it harder for any intervention related changes to be statistically different. If the growth spurt happens during the intervention it may increase its effectiveness, whereas if it happens during the maintenance period it may slow down the adaptation loss. By carefully monitoring the maturity indicators it will be possible to understand and estimate how each participant was affected by the maturation process. A second limitation regards the study’s external validity: due to its length and great number of testing and training sessions, only participants and families that are already inclined towards higher levels of PA will likely choose to participate. Since PA levels are being measured objectively, it will be possible to characterize and compare this study with others and check this assumption.

## Data Availability

Not applicable.

## Abbreviations

3D: Three-Dimensional
6MWT: Six Minutes Walking Test
CIBR: Centre For Interdisciplinary Brain Research
CP: Cerebral Palsy
DSRT: Dynamic Stretch Reflex Threshold
EMG: Electromyography
GMFCS: Gross Motor Function Classification System
GMFM: Gross Motor Function Measure
HPI: Head Position Indicator
H-Reflex: Hoffman Reflex
MEG: Magnetoencephalography
MG: Medial Gastrocnemius
MVC: Maximum Voluntary Contraction
NMRC: Neuromuscular Research Center
PA: Physical Activity
PAD: Post-Activation Depression
RCT: Randomized Controlled Trial
RMS: Root Mean Square
ROM: Range of Motion
S1: Testing Session One
S2: Testing Session Two
S3: Testing Session Three
Sol: Soleus
TA: Tibialis Anterior
TD: Typically Developing
TSRT: Tonic Stretch Reflex Threshold
